# Stakeholder Perspectives on Built Environmental Factors to Support Stroke Rehabilitation and Return to Everyday Life

**DOI:** 10.1111/hex.70339

**Published:** 2025-07-02

**Authors:** Laila de Vries, Maya Kylén, Tony Svensson, Jodi Sturge, Ruby Lipson‐Smith, Steven M. Schmidt, Hélène Pessah‐Rasmussen, Marie Elf

**Affiliations:** ^1^ School of Health and Welfare Dalarna University Falun Sweden; ^2^ Faculty of Health Science Kristianstad University Kristianstad Sweden; ^3^ School of Information and Engineering Dalarna University Falun Sweden; ^4^ Urban and Regional Studies Royal Institute of Technology, KTH Stockholm Sweden; ^5^ Department of Design, Production and Management, Faculty of Engineering Technology University of Twente Enschede the Netherlands; ^6^ The MARCS Institute for Brain, Behaviour and Development Western Sydney University Westmead Australia; ^7^ The Florey Institute of Neuroscience and Mental Health Parkville Australia; ^8^ Department of Health Sciences, Faculty of Medicine Lund University Lund Sweden; ^9^ Department of Neurology, Rehabilitation Medicine, Memory Disorders and Geriatrics Skåne University Hospital Malmö Sweden; ^10^ Department of Clinical Sciences Lund Lund University Lund Sweden

**Keywords:** built environment, home rehabilitation, life after stroke, person‐centred, qualitative method, rehabilitation process, stroke recovery

## Abstract

**Background:**

The transition to undertaking rehabilitation in the home or local neighbourhood calls for an extensive understanding of which aspects of the built environment are important for people with stroke.

**Objective:**

This qualitative study aims to explore how home and local neighbourhood environments support or hinder rehabilitation for people who have had a stroke from the perspectives of various stakeholders.

**Methods:**

Through a purposive selection method, data were collected through semi‐structured interviews with 16 stakeholders: people with stroke (*n* = 3), significant others (*n* = 3), healthcare professionals (*n* = 4), care managers (*n* = 3) and architects (*n* = 3). Content analysis was used to identify patterns and create themes.

**Findings:**

Sixteen stakeholders, including 12 women and 4 men aged 30–74, participated in this study. Our findings identify areas linked to the WHO age‐friendly environment framework, which addresses environmental limitations relevant to stroke rehabilitation. The categories used and factors identified: (1) Outdoor environments: accessibility, safety and supportiveness. (2) Transport and mobility: accessible and reach central services. (3) Housing: adaptations, layout and accessibility. (4) Social participation: spaces that are varied and flexible. (5) Social inclusion and non‐discrimination: shared decision‐making. (6) Civic engagement and employment: supporting environments. (7) Communication and information: digital accessibility. (8) Community and health services: patient‐centred approach and access to varied rehabilitation.

**Conclusion:**

This study brings together multiple perspectives from key stakeholders with experience within stroke care. By integrating insights, these findings highlight how built environmental factors in the home and local neighbourhood can support the transition to home‐based rehabilitation, which can improve recovery and return to everyday life. In turn, this study contributes to the innovative development of home and neighbourhood environments to influence and support stroke rehabilitation. Linking the findings to the WHO framework increases our understanding of a supportive environment for people with stroke, but also for people with other long‐term conditions.

**Patient or Public Contribution:**

This qualitative study is part of a comprehensive research project ‘(Built Environments to support rehabilitation for people with stroke, B‐SURE)’, which aims to investigate how factors in the built environment influence stroke rehabilitation and to develop built environment solutions. B‐SURE has a participatory methodology that essentially includes and involves the stakeholders in the multiple stages of the study and ensures an iterative and collaborative process.

## 1 Introduction

Healthcare is undergoing significant transformation. The shift from centralised institutions to home‐ and community‐based settings is increasingly common for long‐term conditions, such as following the acute phase of illnesses like stroke [1, 2, 3]. This change in healthcare delivery aims to empower individuals and communities by providing equitable, high‐quality healthcare that is accessible, responsive and adaptable to diverse needs, regardless of geographic, socio‐economic or personal circumstances [3]. A key aspect of this vision is the commitment to making healthcare person‐centred and seamlessly integrated into daily life, fostering health as a shared and accessible societal resource. In line with this shift, Early Supported Discharge from hospitals and intensive home‐based rehabilitation have become essential components of modern stroke recovery [4, 5]. Consequently, the design of homes and neighbourhoods is vital in facilitating local healthcare. However, the significance of the environment is often overlooked in home‐based rehabilitation [6]. Hence, there is a need to explore how home and local environments can be designed or modified to support the rehabilitation process. This study addresses this challenge by bringing together perspectives from various stakeholders to explore how they reason around the design of the home and neighbourhood environment to support rehabilitation post‐stroke.

### 1.1 Background

The shift towards community‐based healthcare is relevant for people with stroke, a population often with substantial rehabilitation needs beyond hospital care. Globally, over 15 million people experience a stroke each year, with two‐thirds requiring ongoing support to regain independence and manage daily activities [7]. In Sweden, approximately 25,000 people have a stroke annually, creating a high demand for adequate rehabilitation resources [8]. Stroke can impact individuals' autonomy and functional abilities, leading to long‐term disabilities [9, 10]. A systematic review by Lou et al. [11] showed that individuals express a need to adapt to their new situation and life after a stroke. For many, this means adapting to new circumstances and resources while preserving autonomy and making decisions about their rehabilitation. Facing obstacles to reintegration into society can lead to a decreased quality of life and increased social isolation [9, 12].

Stroke rehabilitation focuses on supporting individuals in regaining abilities, optimising remaining capacities, and promoting independent living [2, 3]. A multidisciplinary team often supports the process, and interventions focus on psychological, social and occupational areas, all tailored to the specific needs of the individual [4]. Inpatient and outpatient care should ensure continuous and comprehensive support throughout recovery. In Europe, rehabilitation often begins with high‐intensity, individualised training in a stroke unit. It extends to the home environment, where municipal healthcare facilitates ongoing services [13, 14].

The built environment, including residential buildings, green spaces, transportation systems and community features like gardens, walkable areas and bike paths, plays a crucial role in stroke recovery [6, 15, 16]. It can support or hinder rehabilitation, functional independence and community reintegration [6, 17, 18, 19]. Environmental barriers at home may restrict daily activities [15], while challenges in public spaces, such as uneven terrain or inaccessible transport, can limit mobility and autonomy [20, 21]. Additionally, strong emotional ties to one's home and neighbourhood shape social connections and lived experiences, making relocation or adaptation complex [22].

The built environment is also central to established rehabilitation practice models, clinical guidelines and urban planning guidelines. However, these are often from a technical perspective, with limited attention to the lived experiences and perspectives of the stakeholders affected by the rehabilitation environment. Thus, incorporating the views of people with stroke, their families, healthcare professionals, architects and community planners is vital for advancing stroke rehabilitation and reaching the goal of inclusive person‐centred services.

This study uses the WHO Age‐Friendly Environments Framework (AFEF) [23] to examine stakeholder perspectives on built environmental factors relevant to stroke rehabilitation. Co‐developed with older adults across Europe, AFEF is part of a global movement to guide policymakers in creating supportive environments for people with disabilities and promoting active, healthy ageing [23]. However, existing frameworks primarily address ageing and disability in general, leaving a gap in stroke‐specific guidance.

Rooted in the concept of person–environment fit [24], AFEF emphasises the need for environments to adapt to individuals' evolving needs. It integrates universal design principles to ensure accessibility for all, regardless of ability [23]. By providing key indicators, AFEF offers a structured approach for policymakers and planners, informing decisions on neighbourhood walkability, public transport access and opportunities for social and cultural engagement—critical factors in fostering autonomy, accessibility and inclusivity.

**Figure 1:**
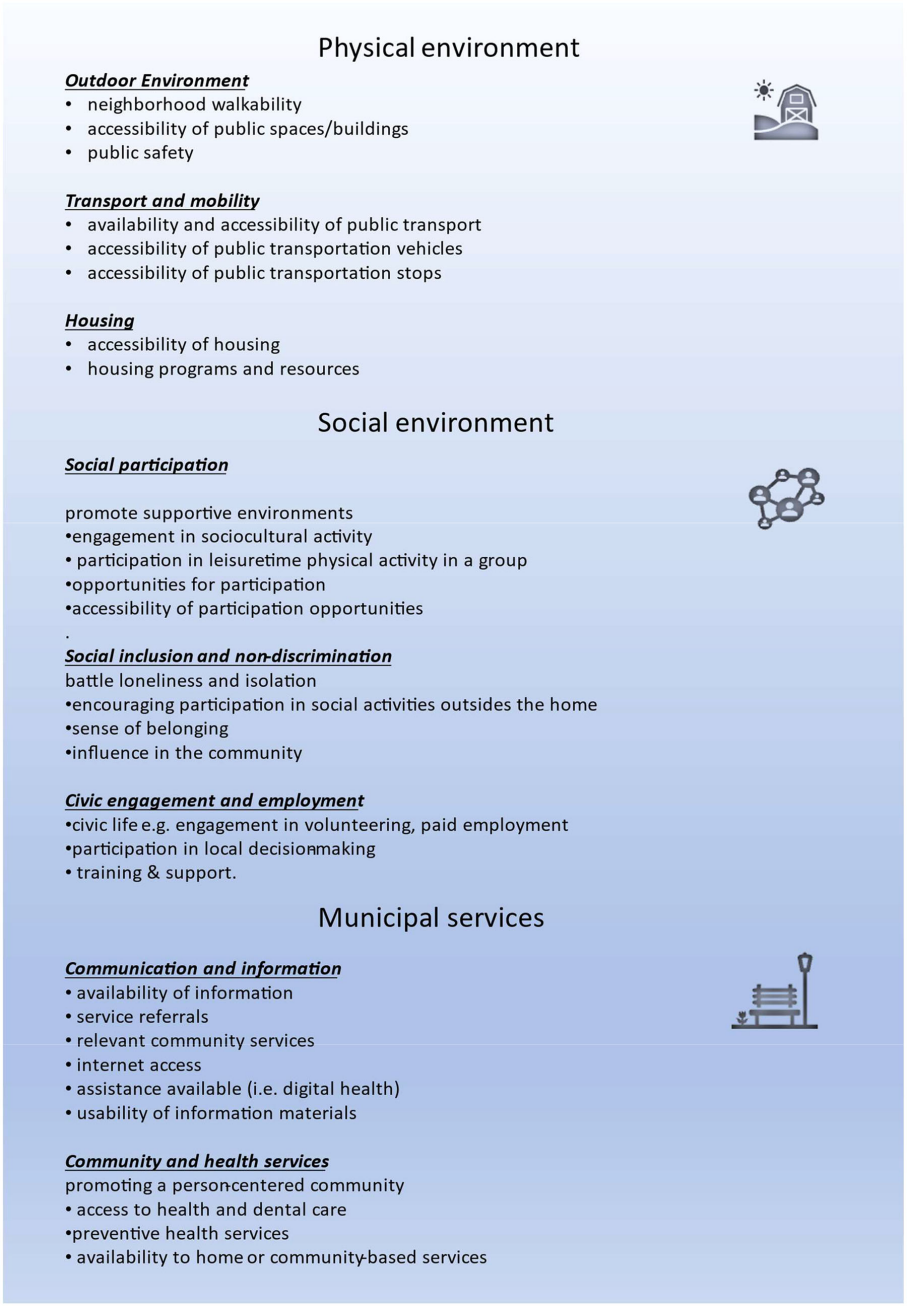
Target topic adapted from the WHO framework [19].

AFEF comprises eight categories, with three overarching areas: the Physical Environment, Social Environment, and Municipal Services (Figure 1). Each domain includes specific target topics previously used as assessment indicators to support practice and policy development [23, 25].

#### 1.1.1 Objectives and Research Questions

In this study, we explore how home and local neighbourhood environments support or hinder rehabilitation for people who have had a stroke from the perspectives of various stakeholders based on the following research questions:

- What factors in the home and neighbourhood environment do stakeholders identify as supporting or hindering stroke rehabilitation and a return to everyday life?
- How do stakeholders envision an ideal, innovative environment that enhances the rehabilitation process?

## 2 Methods

We conducted a qualitative study using in‐depth interviews. The Consolidated Criteria for Reporting Qualitative Research (COREQ) guidelines were applied [26]; see Supporting Information.

### 2.1 Participants and Recruitment

A diverse group of stakeholders was invited to participate, including (1) people who have had a stroke, (2) family members, (3) healthcare staff, (4) healthcare managers/coordinators responsible for stroke rehabilitation in the region and municipalities, and (5) architects and other designers working within healthcare and residential design.

Recruitment for people who have had a stroke, and their relatives, was conducted through stroke units, outpatient rehabilitation units, the Swedish Stroke Association and social media. Staff and managers with clinical experience in stroke rehabilitation were recruited through the researcher's contacts, email lists and targeted personal emails. We aimed to have a balance between different professions with varied experience. We also recruited architects through our network. A purposive selection procedure was used to ensure a sample with broad knowledge and experience [27].

The main inclusion criteria for participation were that adults had to be from one of the stakeholder groups, live in Sweden, and be able to communicate in Swedish. Participants were excluded under circumstances such as a lack of cognitive ability, making it difficult to participate and answer questions during an interview.

Sixteen stakeholders, including 12 women and 4 men aged 30–74, participated in this study. The people who had a stroke lived either in flats or terraced housing. The people who had a stroke and their relatives were pensioners. Further characteristics of the participants are presented in Table 1.

**Table 1:** Characteristics of participants.

Stakeholder	Born year	Sex	Highest education	Interview after discharge (months)	Marital status
Person with stroke 1	1955	M	Upper secondary school	1	Married
Person with stroke 2	1953	M	High school	10	Single
Person with stroke 3	1949	F	University	6	Single

Stakeholder	Born year	Sex	Highest education	Relationship with stroke person	Marital status
Significant other 1	1953	F	University	Wife	Married
Significant other 2	1951	M	University	Brother	Widower
Significant other 3	1952	F	University	Wife	Married

Stakeholder	Born year	Sex	Highest education	Years with stroke experience	Work setting
Rehabilitation staff 1, OT	1993	F	University	3	Outpatient
Rehabilitation staff 2, PT	1984	F	University	11	Outpatient
Rehabilitation staff 3, OT	1977	F	University	9	Outpatient
Rehabilitation staff 4, OT	1979	F	University	1.5	Municipality

Stakeholder	Born year	Sex	Highest education	Years with stroke experience	Work setting
Healthcare manager 1	1968	F	University	11	Municipality
Healthcare manager 2	1969	F	University	1.5	Municipality
Healthcare co‐ordinator 3	1965	F	University	20+	Municipality

Stakeholder	Born year	Sex	Highest education	Work experience (years)	Type of experience
Architect 1	1955	F	University	30+	Healthcare setting
Architect 2	1992	M	University	3	Housing setting
Architect 3	1964	F	University	20+	Healthcare/housing setting

### 2.2 Data Collection Procedures

Data were collected between August and December 2023. The interviews were conducted by one person in the research team (LdV, Msc), who has extensive experience in stroke rehabilitation and is familiar with stroke care as an occupational therapist. The main interview questions were provided to the participants before the interview so they could prepare or clarify any points beforehand. The interview guide (translated from Swedish to English) is presented in Appendix 1. It was developed based on the expertise and knowledge of the research team, followed by piloted testing and refinement. The interview questions were mainly open‐ended, but at the start of the interview, demographic questions were asked, such as date of birth, effects of stroke, years of experience, marital status, work setting, time elapsed since stroke, education level, work status, type of home, and location in Sweden.

The interviews were conducted via Zoom (*n* = 11), physical meetings in the participants' homes (*n* = 3), or telephone (*n* = 2), depending on what was convenient for the participants or the geographical distance. They lasted between 40 and 69 min. They were audio recorded and manually transcribed verbatim.

### 2.3 Analysis

We employed inductive content analysis [28] to identify patterns and create themes. To ensure reliability, co‐authors Ld.V., M.K. and M.E. independently read through the interviews and field notes several times to familiarise themselves with the data and gain an overall understanding. Next, meaning units were extracted from the data, consisting of relevant phrases or sentences that conveyed essential information regarding the research questions. These meaning units were condensed and grouped into codes, ensuring the interpretations remained close to the participants' original expressions to establish a sense of trust and reality. The codes were iteratively refined and grouped into broader themes through collaborative discussions among the co‐authors. To enhance rigour and credibility, Ld.V., M.K., and M.E. independently reviewed and cross‐checked the coding, supporting the development of a trustworthy thematic structure and ensuring sufficient data saturation. The final number of interviews was assessed based on the principle of data saturation, indicating that no new themes emerged at a later stage of the data collection [29]. Any discrepancies in interpretation were resolved through collaborative discussions, which were continuously documented to ensure a transparent audit trail.

To support the coding and categorisation of the data, we used NVivo software [30]. The codes were grouped into preliminary themes and then reviewed and refined in collaboration with the research team to reach a consensus on broader themes. This process supported a shared interpretation of the data, and to enhance transparency and transferability, a thematic summary is provided in Appendix 2.

In the second phase, we mapped these themes into the key categories of the AFEF [23]. Codes and sub‐themes were continuously discussed and corroborated with the research team throughout the analysis to ensure trustworthiness. Additional supporting quotes are included in Table 2 to illustrate the themes and give voice to diverse participants.

**Table 2:** Key findings related to the AFEF's three overarching areas involving the eight categories with additional supporting quotes.

AFEF dimension	Key findings from the interviews supporting the rehabilitation (inductive themes)	Supporting quotes
	Physical environment	
Outdoor environments	Accessibility and easy‐to‐navigate, safe environment support social interactions	‘*A chair out in the yard, in the parking lot … or a bench. Yes, so you can sit down, because when you're so limited, all your strength is expended when you have to walk and move. So, when you get out there, you're very tired already’ (Person with stroke 3)*.
		‘*On the farms that we have designed, we have also designed that you can plant plants. The residents can help, and yes, they can plant flowers. And we have also made chicken coops in some places so you can go and fetch eggs. Because animals are also, animals are something positive’ (Architect 1)*.
Transport and mobility	Public transportation needs to be close, accessible and reach central services to support mobility and independence	‘*But also, the infrastructure [that is of high importance]. How frequently the buses run in your area so that you will be able to travel to the rehab establishment or elsewhere if you are now able to. The distance to the bus stop should not be too far, regardless of where you live in the city. I think these considerations are essential, and that you consider more from the needs of the citizens, granting them a mandate. And to think about what older individuals want, are there older individuals with some type of disabilities’ (Manager/stroke coordinator 2)*.
		[Explaining the term flexible bus]
		‘*It's, you get to decide the stop a little yourself. It will arrive at, yes, where you live, at that address. And so, you can be driven to town then or any address there.’*
Housing	Adaptations, layout and accessibility, digital solutions and small adjustments can facilitate significant change	‘*Ah, I would just like a little more innovation when it comes to lighting; that would be nice. If it was prepared so that you could be more flexible with the lighting, maybe there could be more sockets or maybe there will be more fixed armatures, so you have good general lighting’ (Architect 3)*.
		‘*You could consider using a tag instead of a key, like automatic door openers, so you can enter, and it automatically locks behind you…. It could be voice‐controlled functions, like turning on the hallway lights, so you don't have to fumble around. But that various functions could be voice‐activated. You can even program them, but then it's tailored to the individual, and there are already possibilities with apps. Then there are some nice features … sometimes it can be challenging to find your way home again. Having a GPS, of course, with the individual's consent, could be an option. We've tested it in different places in Sweden, where a relative can track your whereabouts, or home care service. For that matter’* (Manager/stroke coordinator 2).
	Social environment	
Social participation	Spaces that are varied and flexible, both indoor and outdoor, allow for social participation and can enhance the rehabilitation process	‘*Yes, it is pretty incredible, but we have very good children. We have a summer cottage, just a small terrace, outdoor toilet, and everything. But they have arranged so my husband and I can be there for a week or so during the summer. And we've even been to Crete’ (Significant other 3)*.
		‘*It is actually a little brighter environment in here. And then it is also like this that we have welfare hostesses here, which we can, we can go to every morning or forenoon, where all the tenants meet and have a cup of coffee and talk a little nonsense’* (Significant other 3).
		‘*… hand exercises or balance training in gardening, being able to plant flowers, practising fine motor skills at the same time, precisely, gardening—it could also be a regular walk in an environment you enjoy … going to the grocery store, even if it's a very over‐stimulating environment. But if you enjoy it or going to a specific store, that could also be that type of environment or activity’* (Rehabilitation staff 1).
		‘*We can work on adjusting the room layout to enhance accessibility and safety and capitalise on nature, light, daylight, or something similar. It could be like combining two rooms into one large space, or we can use a partition to separate a zone, or even utilize some form of flexible space like a multifunctional room, something along those lines. These are the things I believe can work when designing, yes’* (Architect 2).
		‘*I think one becomes more open, wanting to do things that make them feel good. And if you can only sit indoors and not get out, I believe the motivation for everything drops quite a bit. If you can go out and have your coffee, maybe you're also motivated to try cooking dinner, even if chopping vegetables is challenging. Or if you can visit a friend, you're also likely to muster the energy to cycle for a while…’* (Rehabilitation staff 2).
		‘*If I live next to a highway with bad air and it's always dangerous, and there is also ice everywhere and it's dark almost all the time and such, then I will never want to go outside. Then I will never increase my walking distance. Or if I will never meet a person, I will never be happy and motivated for a social context that makes you feel better. Look at the Greeks and all the blue zones and such. You have to be able to get out and meet people and be in a context, and you become healthier’* (Rehabilitation staff 3).
Social inclusion and non‐discrimination	Shared decision‐making fosters empowerment in taking an active role in their own rehabilitation and social inclusion	‘*We made a garden of the senses. Where you have to use your senses, and you get to feel, smell flowers. It's the first time I've been involved in doing that… it was together with the staff. It was occupational therapists, physiotherapists, and then over the years I have brought it with me, so the landscape architect that I worked with, she and I have together tried to develop this. And then it is important when you are outside the houses that there is the possibility to drive a wheelchair, that there are no obstacles in the way, but it should still be an appealing environment—smells and birds, water ripple are always positive’* (Architect 1).
Civic engagement and employment	Environments supporting meeting places are important to encourage civic engagement	‘*[When talking about what is missing in the rehabilitation chain]' “… I see that relative support meetings and things like that are very important. We have participated in two: one in (town) and one in (town), with both relatives and sufferers participating…. During these meetings, it hasn't been quiet for a minute, so I think that they are very important, to meet like this. I have asked after every meeting if they want a change or if we should continue, and everyone wants to continue to meet. You just wish you had the energy to have it more often“* (Significant other 3).
	Municipal services	
Communication and information	Accessibility to digital tools and platforms can enhance quality of life and enable remote rehabilitation	‘*There are also IT fixers, which probably came during the pandemic. Just that, yes, but I need help with my computer or my iPad to get in touch, with connecting to Zoom or connecting to Teams or connecting to FaceTime’* (Manager 2).
Community and health services	Critical to have a patient‐centred approach and access to varied rehabilitation services	‘*I can walk over there in the corner, you know; there are sidewalks for wheelchair users that are made so I can go there. [Referring to places in the local area where the participant can do some training.] Then I've had some training here at the playground. There is gravel and grass, which is a little uneven. So, I have been training on walking’ (Person with stroke 2)*.

## 3 Findings

The analysis revealed themes directly related to the three main overarching areas: physical, social and municipal in the AFEF.

### 3.1 Physical Environment

#### 3.1.1 Outdoor Environment

Stakeholders emphasised that accessible outdoor environments, especially within neighbourhoods, are essential to rehabilitation. They noted that easy navigation from one's home and within the local area supports individuals in regaining mobility and independence. Key factors identified included the accessibility and safety of surfaces, the appeal and welcoming nature of the surroundings, and the availability of communal spaces that encourage social interaction. For instance, a rehabilitation staff member suggests planning public spaces in small clusters:

“… When urban planning, to actually make room for … the places that also attract older adults, ensuring easy access to grocery stores, hair salons, coffee shops, proximity to the library, and the pharmacy. Not centralizing everything in one cluster. There should be some small, little branches if possible, so that it draws people out! I think that is good”.

Rehabilitation staff 4

These elements help individuals practice the physical skills needed for daily activities and foster social engagement, both of which are crucial for effective rehabilitation and reintegration into everyday life.

When discussing innovative environments, participants emphasised the importance of flexibility in the environment. Whether referring to the layout of rehabilitation facilities or outdoor environments, participants valued adaptable environments that allow for new solutions and creative approaches to support individualised rehabilitation needs. An example discussed was to involve nature, not only the physical environment as a healing component, but also using nature within the rehabilitation.

#### 3.1.2 Transport and Mobility

Participants highlighted the critical role of accessible public transportation in the rehabilitation process. They emphasised the importance of the availability and physical accessibility of buses and trains and the proximity of stops, ideally within walking distance. Accessible transportation networks are essential for supporting mobility and independence, particularly as individuals recovering from a stroke often face temporary or permanent driving restrictions. Access to public transportation facilitates participation in rehabilitation activities, social engagement and access to essential services, all of which are vital to returning to everyday life and reintegrating into the community.

“It takes more than 3 months to regain a driver's license…. What does it look like with public transport? Are there possibilities to take it [the transport], are there bus stops and such in the local environment, as well as shops? These are also aspects that are important to look at”.

Rehabilitation staff 1

Another innovative idea which was valuable for a participant involved the term flexibility. The example below discusses an adaptable transportation system used in a smaller city in Sweden, which provides flexible transit options tailored to meet diverse accessibility needs, highlighting how adaptable infrastructure can enhance mobility and independence in rehabilitation.

#### 3.1.3 Housing

Participants emphasised the importance of adapting to their home environment in facilitating rehabilitation and returning to daily life. They highlighted the significance of home modifications, such as removing thresholds, widening doors and adding handrails, along with more straightforward adjustments like keeping the space clutter‐free. These adaptations were seen as essential for enhancing accessibility within the home, allowing individuals to navigate important areas and maintain independence. Participants also suggested minor adjustments and solutions which could facilitate positive change and increased independence in daily life, such as improved lighting in the home.

They also discussed the importance of room size, layout and accessibility to aids, which they believe contributes to a supportive environment for rehabilitation. They mentioned that it was essential to create a manageable and encouraging recovery space. The space could not be too big, so it becomes overwhelming, but it provides sufficient open space for safe, effective movement and training.

The participants described the physical environment as not only relevant in itself, but also deeply intertwined with institutional conditions and routines. Delays in receiving timely rehabilitation or prolonged waiting times for housing adaptations were particularly challenging.

“I think, in general, it also depends on where you live in Sweden. I also think about access to various assistive devices, how quickly the different processes for adapting to the environment proceed, and so forth.”

Rehabilitation staff 1

Participants suggested innovative, non‐traditional rehabilitation environments, particularly regarding digital tools within the home. These technologies support daily tasks—such as opening doors, adjusting lighting and controlling various home functions—that may be challenging for individuals in recovery. Additionally, digital tools provide crucial aids, such as reminders, apps and voice commands, which foster independence and enhance cognitive and physical engagement during rehabilitation. These insights suggest that integrating digital tools into the home environment significantly supports independence and recovery, indicating a valuable direction for future rehabilitation approaches.

### 3.2 Social Environment

#### 3.2.1 Social Participation

The participants emphasised that social support plays a crucial role in participation, highlighting the importance of a supportive social environment. They noted that receiving the proper support is essential for rehabilitation, and having the backing of family and friends can be particularly beneficial. Other examples, such as having carers who take a genuine interest in the person's needs or getting to the local store and talking to the staff, are also important. This support can, for instance, increase opportunities for individuals to engage in activities outside the home, thereby enhancing their overall rehabilitation process.

Participants emphasised the role of engaging, safe and harmonious social environments in the rehabilitation process, noting that these spaces contribute to both emotional well‐being and social participation, which are critical aspects of recovery. They identified key environmental factors such as the use of colours, flowers, views of nature, well‐lit and uncluttered spaces, and easy access to personal items that foster a homely and welcoming atmosphere, making rehabilitation more effective by encouraging a sense of security and comfort.

Participants also valued environments that offer private and communal spaces, catering to the need for social interaction alongside personal time for rest and recovery. This balance enables individuals in rehabilitation to engage with family, friends and community members, fostering a supportive network and allowing quiet moments for reflection and healing. In some cases, the home also served as a multifunctional space, enabling work‐from‐home setups that support individuals in maintaining their roles and responsibilities, furthering their sense of autonomy and normalcy during rehabilitation.

“For own well‐being, greenery is nature, so nature is important to humans”.

Person with stroke 3

Some participants emphasised the importance of creating flexible and varied rehabilitation spaces that promote recovery and encourage participation, mainly through social interactions. They highlighted the need for adaptable spaces that respond to diverse needs, including communal areas for group activities, private spaces for individual therapy and layouts that accommodate different room sizes with smooth transitions to facilitate movement and mobility exercises.

Participants also underscored the benefits of exposure to nature and daylight, noting that views of natural surroundings and access to appealing outdoor environments enhance mental well‐being, reduce stress and stimulate recovery. Additionally, they stressed that these settings should prioritise safety and security, offering a calm and predictable atmosphere essential for supporting rehabilitation's physical and psychological aspects.

“Variety is important, yes. Especially if you ask people, where do you feel comfortable? In what environments do you thrive and spend time? The common denominator for these environments is that they are often complex and cohesive. I think it's the same, yes, and it's about finding and being able to orient yourself”.

Architect 3

The participants mentioned that various environmental factors significantly influence their ability to participate in their rehabilitation and return to daily life. They noted that less favourable environments, such as the absence of a ramp or no access to public transport, could lead to social isolation, difficulties with orientation and feelings of insecurity. In contrast, more positive environments, such as proximity to nature or access to the balcony independently, were associated with increased activity levels, greater participation in rehabilitation, the ability to make choices freely, heightened motivation, a sense of well‐being, and more opportunities to rest.

Participants also emphasised that motivation and independence are key elements impacting their participation. They highlighted the importance of thoughtful environmental design within their own homes and in the neighbourhood, access to appropriate adaptations within the person's home, and external support (social network and health services) in facilitating these outcomes and enhancing their overall engagement.

#### 3.2.2 Social Inclusion and Non‐Discrimination

Participants emphasised that shared decision‐making is fundamental to social inclusion. They highlighted the importance of involving users actively in planning processes—whether in designing rehabilitation programmes, shaping services or creating accessible spaces within the community. This involvement is essential for fostering a sense of belonging, promoting equity and empowering individuals to participate in their rehabilitation and social reintegration actively.

“Pedestrian paths, should they be illuminated? In what way can one feel safe in this? And perhaps not just brainstorming on our own but using the citizens, the target group we are addressing. Just as we have asked children about playgrounds, I think it is, and of course, it is very important, but that there is a way to utilize it; one can apply the same concept, even in this target group”.

Manager/stroke coordinator 2

#### 3.2.3 Civic Engagement and Employment

Participants, particularly those with experience of stroke, emphasised the importance of civic engagement, such as volunteering, continuing employment and participating in local decision‐making, as crucial to their ongoing recovery and sense of belonging in society. They noted that involvement in support groups helps maintain social connections and encourages active civic participation, which is vital for rehabilitation and social integration. However, for these activities to be accessible, suitable meeting places, workspaces and social venues within the community are essential. Such environments provide the physical spaces needed for individuals to engage meaningfully, fostering a sense of value, integration and continuity during rehabilitation.

### 3.3 Municipal Services

#### 3.3.1 Communication and Information

Participants described the importance of a digital environment that enables them to stay connected with the outside world from home. They emphasised how crucial it was to access digital tools and platforms that facilitate communication, such as video calls, social media and online communities. Utilising those devices allows them to access essential services, participate in remote rehabilitation programmes and manage daily tasks more independently. For many, these digital tools were vital for enhancing their quality of life, promoting inclusion and supporting their well‐being in a modern, connected world.

“Because if you manage to be preventive, you have to know, and it can be obtained in many ways. But then you also need access to, for example, the internet, and then you need to have broadband or to be able to connect to the net. And it can cost; you need to have a phone or a computer that can handle you being able to go out and seek knowledge”.

Manager/Stroke coordinator 2

#### 3.3.2 Community and Health Services

Participants mentioned the critical need for comprehensive and flexible rehabilitation options tailored to the individual's needs within the home and the community. They stressed the importance of a person‐centred approach to rehabilitation, where services are designed around the individual rather than constrained by rigid societal structures and policies. This approach, they argued, should ensure that community and health services are easily accessible, supporting the individual and providing necessary assistance to their families.

Participants highlighted the importance of accessing various rehabilitation services that can adapt to their specific needs and circumstances, whether it involves in‐home support, community‐based services or specialised health care, including preventive programmes.

“The greenery is good for recovery, and one needs that, I suppose. Also, working with greenery, there are these rehabilitation gardens, right? And then I think that balconies are important there too … you can grow quite a bit on your balcony. It's not just for sitting there and going out, but also being able to work with the green. Cultivation, yes, we should see things grow slowly over time”.

Architect 3

## 4 Discussion

This study explored how home and local neighbourhood environments support or hinder rehabilitation for people who have had a stroke, as perceived by various stakeholders. Referring to the WHO Age‐Friendly Environments Framework, we analysed factors that stakeholders identified as influencing rehabilitation and envisioned innovative solutions for enhancing this process. Such insights are essential for planning and improving diverse environments that support rehabilitation and reintegration into society.

The study participants emphasised the importance of considering person–environment fit in all stages of the rehabilitation process, specifically focusing on accessible indoor and outdoor environments. Hence, a person with physical and cognitive disabilities, which are common after a stroke [7, 10], can be independent and live an active life if the demands of the environment are not too high. This draws attention to the changing nature of the person's abilities after a stroke and the environment. As recently noted by Zhang et al. [31], healthcare staff, designers and policymakers need to be proactive and consider person–environment fit as a relative concept that will change over time. As a person's abilities change or the environment changes, the match gets better or worse, meaning that follow‐ups on the individual's function and environment are essential. Participants who had experienced a stroke highlighted the significance of flexibility in their environments. They stressed the need for adaptable spaces that can respond to changing requirements. This dynamic relationship between individuals and their environments is essential and also aligns with the principles of inclusive design, which advocate for spaces that are accessible, functional and adaptable for people of all abilities. This could be more explicitly included in the WHO framework. By doing so, the framework would more accurately reflect the lived experiences and needs of those requiring long‐term rehabilitation.

The findings highlight that the home is the starting point for activities outside the home, and the participants mentioned the need for accessibility as a chain, moving from one point to another. Physical barriers within the home, such as difficulties with entrance, can prevent individuals from participating in society, even if the outdoor environment is accessible. This finding is supported by many previous studies showing unmet needs related to out‐of‐home mobility among people who have had a stroke [32, 33, 34, 35]. Our participants also emphasised the critical role of transportation as a vital part of the rehabilitation process. Access to public transport was expressed as essential for people who have had a stroke, as many are unable to operate a vehicle safely. This aligns with WHO recommendations for inclusive urban planning, which advocate for accessible, safe and equitable transport systems tailored to different needs. However, even though transportation is imperative for accessing essential places, people who have had a stroke continue to report unmet needs in this domain [33]. Losing independent mobility is frequently linked to decreased community participation, negatively affecting mental health and overall quality of life [36]; even so, public transportation remains under‐represented in research focused on individuals living with the consequences of a stroke [37].

Our participants highlighted the strong connection between the physical and social environment, exemplified by their observation that the absence of a ramp could lead to social isolation. This insight aligns with the WHO framework's emphasis on removing environmental barriers to enhance mobility and prevent social isolation. Acknowledging this actionable link is crucial for healthcare professionals, as social isolation is common among people who have had a stroke and is associated with increased risks of cognitive impairment, cardiovascular disease and mortality [38, 39, 40]. Although comparable studies focusing on environmental factors in similar contexts are limited, a study in rural China found that distant toilets, inaccessible light switches and unsuitable seating were environmental barriers independently associated with social isolation after a stroke [41]. Consistent with the WHO framework's call for universal design, our findings emphasise the importance of creating well‐designed environments that promote physical accessibility and meaningful social connections for people who have had a stroke.

While some studies have suggested that social support after stroke occasionally acts as a barrier as it may reinforce dependency or limit autonomy [22], our study contradicted this. Instead, our findings align with previous research demonstrating the crucial role of social relationships and support networks in promoting independence, facilitating stroke recovery and enhancing the quality of life [42, 43]. In addition, the stakeholders in our study acknowledged how important the digital environment is, both in terms of enabling social contact and being able to participate in rehabilitation programmes remotely. As a narrowing of social networks, fewer social activities and reduced contact with friends are common post‐stroke [43], innovative technologies to overcome such barriers warrant further exploration, which is also acknowledged in the WHO framework.

Interestingly, our stakeholders identified shared decision‐making and person‐centred care as crucial despite not being specifically prompted to discuss this topic. This emerged from various perspectives, including environmental design, such as being involved as a stakeholder when new environments are being built, or in rehabilitation healthcare, such as identifying motivating activities and prioritising based on individual preferences. The WHO framework advocates for participation but could go further in detailing how to operationalise stakeholder involvement in creating age‐friendly environments, particularly for vulnerable populations like people who have had a stroke. Such an approach can help people with a stroke feel involved rather than informed, as shown in previous research [6, 15].

The findings highlighted a gap in rehabilitation. Stakeholders felt that the current system is often constrained by rigid structures that fail to accommodate the individual's development and needs. Participants stressed the need to shift towards a truly person‐centred approach, where rehabilitation is tailored to the individual rather than fitting individuals into standard programmes. Therefore, considering the built environment in rehabilitation planning is crucial. Otherwise, rehabilitation interventions risk being incomplete and less effective. Integrating environmental factors into rehabilitation improves patient outcomes and puts necessary pressure on urban planners and communities to design more inclusive and supportive environments. The experience of rigid and programme‐led rehabilitation has been reported previously [6, 44, 45]. The shift aims to improve access to services and remove barriers to recovery, likely reducing stress for individuals and their families. Data from the interviews and other studies [46] indicate a need to rethink the design and delivery of rehabilitation services and help ensure that environmental factors are considered and support recovery for many individuals [47, 48].

Our study aligns with previous research suggesting that region‐specific strategies are needed to effectively implement home‐based stroke rehabilitation in daily practice [48]. While we did not conduct an in‐depth analysis of that study, our findings similarly highlight the importance of adapting rehabilitation approaches to local conditions and built environments to ensure more effective and person‐centred care.

### 4.1 Strengths and Limitations

A core strength of this study is the inclusion of a diverse group of stakeholders. By discussing the environment and rehabilitation after a stroke from different perspectives, we gained a more comprehensive understanding of the multifaceted challenges and opportunities in designing inclusive and supportive environments for people with a stroke. In addition, the people who have had a stroke were relatively healthy with limited stroke‐related disabilities. Future studies exploring perspectives from a group with more severe disabilities are thus warranted, especially since the demands in the environment will likely have a more significant effect on their everyday life. Finally, using the framework for Age‐Friendly Environments has both strengths and limitations. The comprehensive physical, social and municipal environmental scope aligns well with our study's aim. However, the framework is not well adapted to explore the dynamic interplay between these domains and how rehabilitation can best be supported after a stroke. The focus is firmly on community environments, and therefore, the framework does not adequately address the role of healthcare settings and rehabilitation environments, which were central to our findings.

## 5 Conclusions

This study brings together multiple perspectives from individuals who have experienced a stroke, their family members, rehabilitation physicians, care managers and architects. By integrating the insights of these key stakeholders, the study contributes to the innovative development of home and neighbourhood environments that better support stroke rehabilitation. Our findings highlight critical environmental factors that influence rehabilitation in the home and the surrounding community. While grounded in stroke rehabilitation, these insights can be translated to other clinical contexts and inform the co‐creation of design prototypes for accessible living spaces.

In addition, knowledge of the role of the built environment in stroke rehabilitation can inform the transition to home‐based care, which can improve quality of life and recovery outcomes in the long term. The WHO age‐friendly framework further emphasises the need for accessible, adaptable environments that support individuals with different physical and cognitive abilities.

## Author Contributions

**Laila Vries:** conceptualisation, investigation, writing – original draft, validation, writing – review and editing, formal analysis, data curation. **Maya Kylén:** conceptualisation, funding acquisition, writing – original draft, methodology, validation, visualisation, writing – review and editing, formal analysis, project administration, data curation. **Tony Svensson:** conceptualisation, funding acquisition, writing – review and editing, formal analysis. **Jodi Sturge:** conceptualisation, writing – review and editing, formal analysis. **Ruby Lipson‐Smith:** conceptualisation, funding acquisition, writing – review and editing, formal analysis. **Steven M. Schmidt:** conceptualisation, funding acquisition, writing – review and editing, formal analysis. **Hélène Pessah‐Rasmussen:** conceptualisation, funding acquisition, writing – review and editing, formal analysis. **Marie Elf:** conceptualisation, funding acquisition, writing – original draft, methodology, validation, visualisation, writing – review and editing, formal analysis, project administration, data curation.

## Ethics Statement

Ethical approval from the Swedish Ethical Review Authority (DNR: 2023‐02337‐01) has been obtained for the study.

## Consent

Informed consent was acquired from participants before the interviews. Written information on the study purpose and what would be involved in participating was given before the informed consent. The written information also included confidentiality aspects and their right to withdraw from the study at any time.

## Conflicts of Interest

The authors declare no conflicts of interest.

## Supporting information

Supporting information Interview paper.

ISSM COREQ Checklist.

## Data Availability

The data that support the findings of this study are available from the corresponding author upon reasonable request and anonymised before dissemination.
