# Supplementary material for: Stakeholder Perspectives on Built Environmental Factors to Support Stroke Rehabilitation and Return to Everyday Life
Source: Health Expect. 2025 Jul 2;28(4):e70339. doi: 10.1111/hex.70339 (PMC12215816; doi:10.1111/hex.70339)
Supplement: Supplementary file 1 — Supporting information Interview paper. [file HEX-28-e70339-s001.docx]

## Appendix

### Appendix 1. Interview guide

| **Questions for the person with stroke and relatives** | **Prompts** |
| --- | --- |
| Tell me about your daily routine. What does a typical day look like for you? | Talk about the activities you do in addition to your daily routine. What are your hobbies? |
|  | Tell us about some activities in your everyday life that you find challenging. |
| Can you give examples of factors in the home environment that can support rehabilitation and the return to everyday life? Both at home and in the immediate environment (indoors and outdoors)? | Why do you think these factors are important?"  In what ways do you think the design of the physical environment, both indoors and outdoors, can affect the rehabilitation process of someone who has had a stroke?"  Can you describe what a 'stimulating environment' means to you, and how do you think it can contribute to the rehabilitation process after a stroke?"  Are there any negative and hindering factors? |
| What obstacles or conditions do you see as potential challenges when it comes to creating a supportive rehabilitation environment, and how do you think they can be overcome? | Can you give examples? |
| If you had free rein to change your home to promote rehabilitation after a stroke, what would you change or absolutely want to keep? | Why? |
| If you had free rein to change your immediate environment to promote rehabilitation after a stroke, what would you change or absolutely want to keep? | Why? |
| If you could think freely, how would you like the environment to be designed to facilitate rehabilitation after a stroke? | Which places do you think would be important to include and how would you like them to be designed?  Why are these important?  If you were allowed to think completely outside the box, is there anything missing in the rehabilitation chain? |

| **Questions for architects** | **Prompts** |
| --- | --- |
| Can you describe the considerations you make when planning or designing environments to facilitate rehabilitation after a stroke? (or other complex illness requiring rehabilitation) | Which aspects of home and neighbourhood do you consider to be most important in supporting rehabilitation and return to everyday life?  Why?  How do you think the design of the physical environment, indoors and outdoors, can affect the rehabilitation process of someone who has had a stroke?"  Can you describe what a 'stimulating environment' means to you in the context of rehabilitation, and how can it contribute to the rehabilitation process? |
| What challenges have you encountered in creating supportive rehabilitation environments, and how have you dealt with these? | What are the most tangible obstacles or limitations you encounter when trying to create a supportive rehabilitation environment, and how do you think they can be overcome? |
| If you could make a few changes to an individual's home to promote rehabilitation, what would you change or absolutely want to keep? | Why? |
| If you could make any changes in an individual's immediate environment to promote rehabilitation, what would you change or absolutely want to keep? | Why? |
| If you could think freely, how would you like the environment to be designed to facilitate rehabilitation after a stroke? What places do you think would be important to include, and how would you like them to be designed? | Why?  Any innovative suggestions/ideas? |

| **Questions to staff** | **Prompts** |
| --- | --- |
| Can you describe what a typical working day looks like for you as a rehabilitation professional? | What challenges do you often face in your work to support people who are rehabilitating at home? |
| Based on your experience, what factors in the home and local environment (indoors and outdoors) do you think are most important to support rehabilitation and the return to everyday life? | How do you think the design of the physical environment can affect the rehabilitation process of someone who has had a stroke?"  What does a 'stimulating environment' mean to you in the context of rehabilitation, and how do you think it can contribute to the rehabilitation process?" |
| If you could make some changes to a patient's home to promote rehabilitation, what would you change or absolutely want to keep? | Can you give some examples?  Have you encountered any negative or hindering factors when working on creating a supportive rehabilitation environment? |
| If you could make any changes in a patient's immediate environment to promote rehabilitation, what would you change or absolutely want to keep? | Can you give some examples?  Have you encountered any negative or hindering factors when working on creating a supportive rehabilitation environment? |
| If you could think freely, how would you like the environment to be designed to facilitate rehabilitation after a stroke? | Which places do you think would be important to include and how would you like them to be designed?  Why?  If you were allowed to think completely outside the box, is there anything missing in the rehabilitation chain? |

| **Questions for Managers** | **Prompts** |
| --- | --- |
| What challenges do you face as a rehabilitation manager, especially with regard to creating supportive environments for rehabilitation? |  |
| What aspects of home and community environments do you consider to be most important to support rehabilitation and return to everyday life for your patients? | Why?  How do you think the design of the physical environment might affect the rehabilitation process of someone who has had a stroke?  What does a 'stimulating environment' mean to you in the context of rehabilitation, and how can it contribute to the rehabilitation process?" |
| As a director of rehabilitation, what obstacles or limitations do you often encounter when it comes to creating a supportive rehabilitation environment, and how do you think they can be overcome? |  |
| If you had the opportunity to influence the design of a patient's home to promote rehabilitation, what would you like to change or absolutely want to keep? | Why? |
| If you had the opportunity to influence the design of a patient's immediate environment to promote rehabilitation, what would you like to change or absolutely want to keep? | Why? |
| If you could think freely, how would you like the environment to be designed to facilitate rehabilitation after a stroke? | Which places do you think would be important to include and how would you like them to be designed?  Why?  If you were allowed to think completely outside the box, is there anything missing in the rehabilitation chain? |

### Appendix 2. Table of emerged categories and subthemes

| WHO categories | Subthemes identified | Description of the subthemes | Quotes |
| --- | --- | --- | --- |
| **Outdoor Environment**  *Target topics:*  *neighborhood walkability, accessibility of public spaces and buildings* | **Accessibility in neighbourhood** | Encompasses the ability to navigate important areas within one's home and local surroundings. Also include local area navigation, focusing on accessible surfaces, inviting surroundings, and the availability of communal spaces. | “a chair out in the yard, in the parking lot, so you can sit down, or a bench.... Yes, so you can sit down, because when you, you know when you're so limited, all your strength is expended when you have to walk and move. So, when you get out there, you're very tired already” (Person with stroke 3).  ” But maybe it's precisely that, to reconsider... I'm thinking when urban planning, to actually make room for those small... the places that also attract the older adult, that there is easy access to it, like grocery stores, hair salons, coffee shops, proximity to the library, and the pharmacy. Not centralizing everything in one cluster. There should be some small, little branches if possible, so that it draws people out! I think that is good” (Rehabilitation staff 4).  ” Well, there's a lot there [local environment], yes, but everything from how, well, everything looks when you step outside the door, that, well, that it should be easily accessible for walking, whether it's a courtyard or a garden. There are limitations there, like gravel or having a lawn. So, I think it's important to also review those aspects” (rehabilitation staff 1).  “I believe it's different, but it depends on the facilities and the green spaces around. I mean, the city is good, but it should be accessible, like within walking distance from home to common facilities: a playground, a green park, a square, or something like a grocery store. But, of course, the countryside offers something different, like silence, tranquility of some sort. However, it can also be challenging because, in rural areas, it might be difficult, harder to socialize, to integrate into the community, perhaps because houses are far apart or something like that” (Architect 2).  “That it feels inspiring and safe to go outside. I'm thinking about both how to get out of your home, how stairs and entrances are designed, and then what you encounter once you're outside. Do I end up in a parking lot, or do I come out into a small backyard with some sofas and plants, or" (rehabilitation staff 4). |
| **Transport and mobility**  ***Target topics:***  *availability and accessibility of public transport, accessibility of public transportation vehicles, accessibility of public transportation stops* | **Accessible transportation** | Encompasses quotes emphasizing the significance of transportation networks for mobility in society. Local modes of transport enable access to various establishments and social contexts. | “It takes more than 3 months to regain a driver's license. To make sure that, what does it look like with public transport? Are there possibilities to take it [the transport], are there like bus stops and so in the local environment, as well shops. These are also aspects that are important to look at” (Rehabilitation staff 1).  ” But also, the infrastructure [that it is of high importance]. How frequently the buses run in your area, so that you will be able to travel to the rehab establishment or elsewhere if you now are able to. The distance to the bus stop should not be too far, regardless of where you live in the city. I think these considerations are essential, and that you consider more of the needs of the citizens, granting them a mandate. And to think about what older individuals want, are there older individuals with some type of disabilities” (Manager/ stroke coordinator 2). |
| **Housing**  ***Target topics:***  *accessibility of housing, housing programs and resources* | **Adaptations and modifications** | Encompasses areas such as home modifications, assistive devices, simpler adjustments within the home and keeping the space clutter-free. Home modifications may involve actions like removing thresholds, widening doors, adding handrails etc.  Facilitates rehabilitation and the return to daily life. Assistive devices that can be borrowed from the municipality or purchased and should be available upon returning home. | "If he's in the apartment, he can just roll out; there's a small threshold to the apartment, and then the elevator is right there. He can press the button, go down, and when he arrives, he just glides out. There is a slight incline downward, and there is a button there. When pressed, both doors open. So, it's not a problem, and from the other side, he has one of these tags, or whatever you call it, a key fob. Pressing it opens the gate, and then he can enter" (Significant other 2). |
|  |  |  | "I actually think it's crucial, regardless of whether you're ill, but especially if you've had a stroke, for example have clean surfaces. There shouldn't be too many rugs, and there shouldn't be too many cables. For me, that is a harmonious home, that impacts both physical and mental health. It allows both him and me to feel more relaxed and not constantly think about..." (Significant other 1). |
|  | **Accessibility in the home** | Encompasses the ability to navigate important areas within one's home.  Emphasizing factors like room size, layout, accessibility aids, and home adaptations for independence. Also include local area navigation, focusing on accessible surfaces, inviting surroundings, and the availability of communal spaces.  This also involves aspects of daily life in terms of safety, related to the built environment. | "If it's a person who uses a wheelchair, one would want to assess the physical environment and ensure that it is accessible everywhere. This includes both entering and exiting the residence, navigating all parts of their home, and being able to manage their daily life" (manager/stroke coordinator 3).  “I would probably remove all carpets and cords that are in the way so that you don't risk falling when walking around. Remove the curtains so that some light comes in for better visibility. Yes, I think an open floor plan is good if they have the opportunity for gait training, being able to walk around without it being tight when turning, and having spacious bathrooms is good so that you can move around easily, if you need to get into the shower or to make is easier to move about, lots of space" (Rehabilitation staff 2).  “That is a really important thing, security is, security and orientation and visibility [referring to what you see in a room/space]. That's what I mean by how to see where to go, if you are able to catch the interest by something. And there is clearly security in your home environment where you have always been, then you feel more secure than when you come to a completely foreign environment” (architect 1) |
|  | **Equal care within the community** | Thoughts are raised about the differences across Sweden regarding stroke care. The challenges of having different principles that can complicate the overall rehabilitation process. The consequences may include individuals who do not receive rehabilitation when required or extended waiting time for housing adaptations. Additionally, it's emphasized that training has associated costs, posing an economic challenge for some. It's crucial that the entire rehabilitation chain functions properly, including timely handover reports. | " I think, in general, it also depends on where you live in Sweden. I also think about the access to various assistive devices and how quickly the different processes for adapting the environment proceed and so. There are quite significant differences across Sweden, so the wish is also that it should be more easily accessible and that the rules should be the same for everyone, regardless of the region you belong to" (Rehabilitation staff 1).  “But the important thing is the collaboration, that the whole chain works, from the handover, whether it's from inpatient care to, whether it's then to short-term care or directly to us [in the community]. That it doesn't take too long there” (Manager/stroke coordinator 2). |
|  | **Digital environmnet** | Describe an environment that incorporates the use of digital tools, thus technology can be employed to ease daily tasks like opening doors, controlling lights etc. | *“You could consider using a tag instead of a key, like automatic door openers, so you can enter, and it automatically locks behind you. Oh, one could have, it could be voice-controlled functions, like turning on the hallway lights, so you don't have to fumble around. But that various functions could be voice-activated. You can even program them but then it's tailored to the individual, and there are already possibilities with apps. Then there are some nice features that you test sometimes, now I'm back to the cognitive aspect. Sometimes it can be challenging to find your way home again. Having a GPS, of course, with the individual's consent, could be an option. We've tested it in different places in Sweden, where a relative or can track your whereabouts, or home care service. For that matter" (Manager/stroke coordinator 2).*  *"Many patients find it very difficult that home care comes to their home, but some patients can also find it quite convenient, so it can also limit them, because they get help with it, so they may not feel the same need to train on these activities and become independent in them. So, there I'm thinking, probably a robot that can both encourage but also help with the practical aspects so that one can still feel more independent” (Rehabilitation staff 1).* |

| WHO domain | Subthemes identified | Description of the subthemes | Quotes |
| --- | --- | --- | --- |
| **Social Participation**  *Target topics:*  *engagement in sociocultural activity, participation in leisure-time physical activity in a group, opportunities for participation, accessibility of participation opportunities* | **Engaging environment** | Describes factors such as creating an environment that is homely, harmonious, safe, and stimulating. Examples on contributing aspects are: colors, flowers, having access to a view, proximity to nature, a well-lit environment, uncluttered spaces, easy access to belongings, access to private and social spaces.  When describing a stimulating environment, it is characterized by spaces that evoke creativity, engage different senses, instill a sense of security, offer a varied and flexible environment, welcoming atmospheres, quiet places, and spaces adapted to the individual. These environments are associated with nature, animals, activity-based training places that facilitate social activities.  Here, participants discuss places that are important to them, which appear to be highly individual. They talk about places that are diverse, providing opportunities in both the community (with, for example, family and friends) and personal time. A place in the home that is tailored to an individual. A place that allows for stimulation and recovery, which can include both the flexible and stimulating environmental factors.  The concept of the home as a workplace has also been mentioned in this category. | "A stimulating environment is indeed one that can awaken creativity in patients. When I think of a stimulating environment, I think of elements that can be captured by the different senses. I think that it should be a natural setting that evokes both calmness and, well, when I think of it, I think of a lot of colors, but above all, an environment that stimulates creativity and a desire to engage, find things motivating and enjoyable. That's probably it, precisely that it sparks creativity, motivation, and that it should feel enjoyable" (Rehabilitation staff 1).  "Yes, but this, well, hand exercises or balance training in gardening, being able to plant flowers, practicing fine motor skills at the same time, precisely, gardening—it could also be a regular walk in an environment you enjoy. But it could also be going to the grocery store, even if it's a very overstimulating environment. But if you really enjoy it or going to a specific store, that could also be that type of environment or activity" (Rehabilitation staff 1).  ” The shared space is also important, like in terms like people need connected to more, like, need to like feel they’re in the community. That's connected to like the community space like going to the park or into the city and feeling people around” (Architect 2).  “On the farms that we have designed, we have also designed that you can plant plants. That the residents can help and yes plant flowers and such. And we have also made chicken coops in some places so you can go and fetch eggs. Because animals are also, animals are something positive” (architect 1)  “I: What is a stimulating environment for you?  P: some greenery around and benches on which you can sit. Some flowers around too, and that we have. We also have benches, and we have greenery, yes, we even have a park....  I: So, greenery and nature, stimulate do you think?  P: yes, I think it's very important.  I: And why is that important, do you think?  P: for own wellbeing, greenery is nature, so nature is important to humans” (Stroke 3)  "So, different options, everything from quieter places and maybe something to sit on if you want to go to your inner self and do a bit more mindfulness or something like that. But also places to come together, more social interaction, that’s what I’m thinking about. It doesn’t have to be watching screaming children on playgrounds, but it can be, I don’t know, in the vicinity of maybe a convenience store, where many people gather, everyone has to go there, young and old occasionally. Or the local library might be something like that, but that there are places to meet naturally around places in the neighborhood that still attract people, I think would stimulate rehabilitation" (Rehabilitation staff 3)  "I: What do you think is an important place for an individual?  Participant: I think that if I lived in the city and were older, I would like to have the opportunity to go out in some way where there is a park or nature, and to be able to meet others. Church, library, whatever it may be" (Rehabilitation staff 4).  “(When answering about important places) The conservatory is winter adapted, so it is used all year round. And we often sit there, there is a transparent roof, so to speak, so that in the winter you still get sun in, when the sun is shining, it is very bright” (Person with stroke 1) |
|  | **Flexible and varied environment** | Incorporates thoughts on how to design spaces to promote rehabilitation through having flexible solutions and options for a varied environment when looking at communal spaces, private areas, layout, room size, transitions between rooms, views of nature, exposure to daylight, appealing outdoor environment.  Includes aspects such as efficiency, safety, security, a calm atmosphere. | "We can work on adjusting the room layout to enhance accessibility, safety, and also capitalize on nature or light, daylight, or something similar. It could be like combining two rooms into one large space, or we can use a partition to separate a zone, or even just utilize some form of flexible space like a multifunctional room, something along those lines. These are the things I believe can work when designing, yes" (Architect 2).  "And above all, the needs always change for someone in the household, often that's the case. So, I think that, well, overall, it's a good principle to just think flexibly. Can you modify the residence, change the layout, adjust the lighting to control visibility, so you can avoid unwanted attention if it bothers you? For example, if someone is sensitive to privacy, feeling depressed, or something. You need to be able to regulate or adapt" (Architect 3).  "Variety is important; yes, I really believe that. Especially if you ask people, where do you feel comfortable? In what environments do you thrive and spend time in? The common denominator for these environments is often that they are both complex and cohesive. I think it's the same, yes, and it's about finding and being able to orient yourself" (Architect 3). |
|  | **Environmental impact on wellbeing and participation** | In this category individuals describe that different environmental factors affect participation. Less positive environments can cause social isolation, difficulty with orientation, and insecurity. Better environments contribute to increased activity, participation in rehabilitation, option of free choice, increased motivation, sense of well-being, and increased opportunities to rest, among other things. | "And besides, since it's not always necessary for patients to engage in direct social interactions, it can be an alternative and not direct interaction, like going to the park. When they are in public, they can feel like the people around, and that can help them connect more with the neighborhood…. And a lot is about health and well-being. I found that the location of the house, the surroundings, and the distance between the residence and the city square and the nearby park are very, very important. It should be accessible to patients for daily routines." (Architect 2). |
|  |  | - Motivation   This part of the subtheme highlights that motivation is essential to participate in activities, and the design of the environment, adaptations, and external support are areas of significance to facilitate this.   - Independence   Here the participants describe how the design of the environment, adaptations, and external support are factors that can either increase or hinder independence. | “I'm just thinking of a patient I have, she has a pretty big balcony, and she does a lot of work with her flowers. But she has a tough time, she must have something and hold on to. But she cannot get out with her walker, and she has another walking aid there without wheels. But it is difficult to bring a walking aid and a watering can with water in it” (Rehabilitation staff 4)  “I think one becomes a bit more open, wanting to do things that make them feel good. And if you can only sit indoors and not get out, I believe the motivation for everything drops quite a bit. If you can go out and have your coffee, maybe you're also motivated to try cooking dinner even if chopping vegetables is challenging. Or if you can visit a friend, you're also likely to muster the energy to cycle for a while or, well, yes." (Rehabilitation staff 2).  “If the outdoor environment is, if I live next to a highway with bad air and always dangerous and there is also ice everywhere and dark almost all the time and such, then I will never want to go outside. Then I will never increase my walking distance. Or if I never meet a person, I will never be happy and motivated for a social context that makes you feel better. Look at the Greeks and all the blue zones and such. You must be able to get out and meet people and be in a context and you become healthier” (Rehabilitation staff 3).  “The public transport, that is, the transport part with, yes with bus stops and so nearby, plays a big part in how independent a person can be or not, but then also of course the entire home [referring to important places]” (Rehabilitation staff 1)  “I: outdoors what factors do you think about there, what is most important there when about to support them?  P: yes, it could be if there is someone who has a lot of level differences out there, to get a ramp so you can get out of the house or maybe asphalt the road so that you can walk easier with a walking aid” (Rehabilitation staff 2) |
|  | **Social support** | This category involves the social environment and its importance to participation. It is essential that the individual receives the right support. It appears to be beneficial for an individual to have support from family and friends (i.e. increased opportunities to access activities outside home). | "What is important for the patient but somehow enable it to be functional so that I'm thinking here about accessibility to get the right aids but also to get that, like, physical assistance from another person, whether it's a relative or, for example, home care, but just that it's very important to ensure that there are resources, both socially but above all physically so that it, yes, should facilitate patients in being able to do such important things." (Rehabilitation staff 1).  “Yes, it is pretty incredible, but we have very good children. We have a summer cottage, none, just a small terrace, outdoor toilet and everything. But they have arranged so my husband and I can be there for a week or so during the summer. And we've even been to Crete” (Significant other 3)  “I: can you give me examples of how you feel it is more positive (referring to their senior housing)?  P: yes, it is actually a little brighter environment here. And then it is also like this that we have welfare hostesses here, which we can, we can go to every morning or forenoon, where all the tenants meet and have a cup of coffee and talk a little nonsense” (Significant others 3) |
| **Social Inclusion and non-discrimination**  Target topics  -Sence of belonging | **Shared decision making** | Emphasizes the importance of involving the target group in planning, whether it concerns rehabilitation or the design of services/places in society. | "Pedestrian paths, should they be illuminated, in what way can one feel safe in this. And perhaps not just brainstorming on our own but actually using the citizens, the target group we are addressing. Just as we have asked children about playgrounds, I think it is, and of course, it is very important, but that there is a way to utilize it, one can apply the same concept, even in this target group" (Manager /stroke coordinator 2).  “We made a garden of the senses. Where you have to use your senses and you get to feel, smell flowers. It's the first time I've been involved in doing that. And it was, it was together with the staff. It was occupational therapists, physiotherapists and then over the years I have brought it with me so the landscape architect that I worked with, she and I have together tried to develop this. And then it is important when you are outside the houses that there is the possibility to drive a wheelchair, that there are no obstacles in the way, but it should still be an appealing environment, smells and birds, water ripple are always positive” (Architect 1) |
| **Civic engagement and employment**  **-** *engagement in volunteering, paid employment*  *- participation in local decision-making -training & support* | **Shared decision making**  **Social support** | This category appears to have overlapped components from the above co-design subtheme and social support subtheme. In a few interviews engagement in support groups was highlighted to be important in terms of continuing recovery and being a part of society. | [When talking about what is missing in the rehabilitation chain] “But on the other hand, I see that relative support meeting and things like that are very important. We participated in two, one in (town) and one in (town), with both relatives and sufferers participating. And my thought was that you might want to split up relatives and stroke sufferers. And have different groups. But during these meetings it hasn’t been quiet for a minute, so I think that they, and they are very important, to meet like this. I have asked after every meeting if they want a change or if we should continue and everyone wants to continue to meet. You just wish you had the energy to have it more often” (significant other 3) |

| WHO Domain | Subthemes identified | Description of the subthemes | Quotes |
| --- | --- | --- | --- |
| **Communication and information**  *Target topics:*  *- availability of information*  *- internet access*  *- usability of information materials* | **Digital Environment** | Describes an environment that incorporates the use of digital tools at home for communication with the outside world, accessing platforms and utilizing devices such as computers, tablets, and mobile phones. | Because if you are going to manage to be preventive, yes you have to have the knowledge, and it can be obtained in many ways. But then you also need access to, for example to the internet, and then you need to have broadband or to be able to connect to the net. And it can cost, you need to have a phone or a computer that can handle you being able to go out and seek knowledge (Manager/Stroke coordinator 2).  There are also IT fixers, and it probably came during the pandemic. Just that, yes but I need help with my computer or my iPad to get in touch, with connecting to Zoom or connecting to Teams or connecting to Facetime (Manager 2). |
| **Community and health services**  *Target topics:*  *- access to health and dental care*  *- supportive health services*  *- availability to home- or community-based services* | **Rehabilitation options** | Emphasizes the need for tailored rehabilitation both within the home environment and out in the community. A more flexible way of looking on rehabilitation and with a person centred perspective – with the individual in focus rather than being limited to the specific structures of the society and policies. Perhaps looking at new technology or new places (online?). | "The greenery, that it's good for recovery and one needs that, I suppose. Also, working with greenery, there are these rehabilitation gardens, right? And then I think that balconies are important there too because you can have a large one, you can grow quite a bit on your balcony. It's not just for sitting there and going out but also being able to work with the green. Cultivation, yes, we should see things grow slowly over time" (Architect 3)  “I can walk over there in the corner you know, there are sidewalks for wheelchair that are made so, I can go there. [Referring to places in local area where the participant can do some training]. Then I've had some training here at the playground. There is both gravel, there is grass which is a little uneven. So, there I have been training on walking” (Person with stroke 2). |
